# Assessment of toxicity and antimicrobial performance of polymeric inorganic coagulant and evaluation for eutrophication reduction

**DOI:** 10.1038/s41598-024-53714-9

**Published:** 2024-02-09

**Authors:** Marwa Youssef, Sara S. El-Tanany, Yassmin Moatasim, Shimaa M. Abdel Moniem, Bahaa A. Hemdan, Nabila S. Ammar, Gamila E. El-Taweel, Azza M. Ashmawy, Mohamed I. Badawy, Mohamed R. Lasheen, Hanan S. Ibrahim, Mohamed Eid M. Ali

**Affiliations:** 1 Water Pollution Research Department, Environmental and Climate Changes Institute, National Research Centre, El-Buhouth St., Dokki, P.O. 12622, Cairo, Egypt; 2 Environmental and Occupational Medicine Department, Environmental and Climate Changes Institute, National Research Centre, El-Buhouth St., Dokki, P.O. 12622, Cairo, Egypt; 3 Centre of Excellence for Influenza Viruses, National Research Centre, El-Buhouth St., Dokki, P.O. 12622, Cairo, Egypt

**Keywords:** Wastewater treatment, Coagulation process, Antimicrobial agent, Iron-based poly inorganic coagulants, Toxicity, Biochemistry, Biological techniques, Chemical biology, Ecology, Microbiology, Environmental sciences

## Abstract

In this study, the efficacy of the promising iron—based polymeric inorganic coagulant (POFC) was assessed for the reduction of eutrophication effect (freshwater toxicity) and the microbial loads from wastewater. Toxicity assessment for POFC was conducted on mice and skin cell lines. The results confirm the lower toxicity level of POFC. The POFC showed excellent antibacterial efficacy against Gram-positive and Gram-negative bacteria. Moreover, it demonstrated a remarkable effectiveness against black fungus such as *Aspergillus niger* and *Rhizopus oryzae*. Additionally, POFC showed antiviral effectiveness against the highly pathogenic H5N1 influenza virus as well as Middle East respiratory syndrome coronavirus (MERS-CoV) and severe acute respiratory syndrome coronavirus 2 (SARS-CoV-2). POFC-based treatment gives excellent removal percentages for phosphate, and phosphorus at doses below 60 ppm with a low produced sludge volume that leads to 84% decrease in the rate of eutrophication and freshwater toxicity. At a POFC concentration of 60 ppm, remarkable reduction rates for total coliforms, fecal coliforms, and *E. coli* were achieved. After POFC-based coagulation, the produced sludge retains a lower bacterial density due to the antibacterial activity of POFC. Furthermore, it revealed that the observed removal efficiencies for fungi and yeasts in the produced sludge reached 85% at a POFC dose of 60 ppm. Overall, our research indicates that POFC has potential for application in pre-treatment of wastewater and serves as an antimicrobial agent.

## Introduction

Municipal wastewater contains a complex combination of bacteria, suspended sediments, organic chemicals, and nutrients that, if improperly handled, may have serious effects on the environment and human health^1,2^. The excessive amount of these nutrients causes a phenomenon called eutrophication, that should be taken seriously since it may harm aquatic ecosystems and causes difficult problems for a variety of water consumption applications. Eutrophication arises from the buildup of nutrients, especially phosphorus and nitrogen in the form of phosphate and nitrate^3^. The accumulation of these nutrients causes changes in species composition, an increase in toxic algal blooms, and emergence of bottom anoxia^4^. Phosphorus is released into water bodies by anthropogenic sources, such as home and industrial wastewater, detergents, animal waste, and fertilizers^5^. Untreated municipal wastewater may introduce ~ 5–20 mg/L of total phosphorus into the environment which upsets the balance and regular operation of the water bodies^3^. The prevalence and spread of water-borne diseases are considerably influenced by these nutrients. According to several studies, the discharging of wastewater effluents into different ecosystems, such as large lakes, streams, and rivers, may change the bacterial populations. Meanwhile, untreated wastewater may operate as a significant reservoir of dangerous microbes, including viral pathogens^6^, bacteria, and fungus so it increases the risk of waterborne infections^7^.

These infections might be dangerous to the public health since they can spread to people or livestock via direct touch or aerosol inhalation^7,8^. Therefore, it is essential to remove contaminants from wastewater and treat effluents effectively to fulfil standard discharge criteria and protect both the environment and human health^8–10^. Meanwhile, the efficiently treated wastewater can be reused for various purposes, thus achieving the current trends of sustainable development goals^11^.

Traditional municipal wastewater treatment methods have mainly concentrated on the removal of solids and organic pollutants; however, the efficiency of these methods in removing microbial contaminants has not been systematically evaluated^11–14^. New treatment methods have been created to enable rigorous management of water quality and microbiological burdens in order to protect freshwater resources and public health^15–17^. The instability of colloidal suspensions and the removal of suspended particles and organic materials are made possible by the technique of coagulation/flocculation, which is one of these methods. Coagulation–flocculation has been widely applied due to its low economic cost, ease of operation, and high efficiency as well as its applicability during pretreatment, main treatment, and post-treatment^18–22^.

Coagulants divided into three categories: organic, inorganic, and hybrid materials^22^. Conventional inorganic coagulants based on iron (Fe) and aluminum (Al) are the most commonly used ones^19^. However, these coagulants like aluminum sulfate, ferric sulfate, and ferric chloride have substantial downsides on health and environment^21^. These coagulants can release residues like aluminum metal and harmful monomers into the treated wastewater which thought that both have a damage effect on the human nervous system and may have possible link to Alzheimer's disease^23,24^. Also, ferric ions can cause significant alterations in pH of the discharged effluent as well as corrosion of equipment^25,26^. With the continuous changes in the aquatic environment, the demand for effective coagulants is growing. So, a large number of researchers have recently studied and improved coagulation-flocculation techniques by using hybrid coagulants^22^. Many studies proved that inorganic polymeric coagulants (IPCs), like polyaluminum chloride (PAC), poly-ferric sulfate (PFS) and poly-ferric chloride (PFC), exhibit greater coagulation efficiency than the typical inorganic coagulation salts^20–22,27,28^. IPCs have hydrolysis and polymeric characteristics that may postpone the precipitation of hydroxide after dilution, increasing the life of their polymeric forms. Additionally, IPCs often have large positive charges and high molecular weights, which contribute higher neutralizing capability for particulate and colloidal organic matter more than traditional coagulants since these particles in wastewater and water, are typically negatively charged which leading to increase the efficiency of IPCs in wastewater treatment systems over conventional coagulants^20^. Another feature of using IPCs is the less amount of the generated sludge and the ultimate reduction in the cost of purification^29^. Shi et al.^18^ studied the efficiency of using inorganic composite coagulant polyaluminium ferric silicate (PAFS) for treating wool scouring wastewater and compared the results with conventional coagulants, polyaluminium chloride (PAC) and polyferric sulfate (PFS). They found that coagulation performance improved with using PAFS over a wider range of pH (3–7) and higher removal rates were achieved for chemical oxygen demand (COD) (97%) and turbidity (99%)^18^.

Polymeric ferric chloride (PFC) is one of the efficient IPCs that can be used in the coagulation-flocculation process at low doses and fit to use at a wide range of pH and temperature^22^. Abujazar et al.^22^ reported that PFC are widely used during the treatment of different wastewater types, including municipal, industrial, and landfill leachate and achieved removal efficiencies for COD, total organic carbon (TOC), and color ranged from 46 to 100%. Although the coagulation efficiency of using these coagulants is well-proven, their efficiency in removing microbes is not well documented. It is commonly acknowledged that there is an urgent need for efficient treatment methods that may reduce harmful microbial contamination while still being simple, inexpensive, and broadly applicable^8,13,30–32^.

In the view of above discussion and taking the economic cost into account, low cost raw materials (waste) were exploited to synthesize a coagulant POFC-1-0.8 and it subjected to detailed characterization. The antibacterial, antifungal and antiviral activity of the coagulant was assessed and to best of our knowledge, this has not been addressed in the previous studies. Moreover, toxicity impact of the applied POFC was assessed. The current research focuses on employing feasible treatment way for reduction of eutrophication and freshwater toxicity as well as microbial decontamination of municipal wastewater. Therefore, the goal of this research is to estimate the toxicity of POFC and its antimicrobial activity. Moreover, the effectiveness of POFC coagulant for municipal wastewater treatment was studied particularly in terms of lowering microbial loads in treated effluent and in the generated sludge as well as declining eutrophication impact of treated effluent on surface water. The results are promising to enhance the production and large-scale application of POFC.

## Materials and methods

### Preparation, and characterization of POFC coagulant

POFC was synthesized as detailed in our earlier publication by Ali et al.^33^. A comprehensive characterization of these polymeric coagulants can be found in our previously published research with selection of POFC-1-0.8 as an optimum coagulant, as previously reported in Ali et al.^33^.

### Toxicity characteristic of inorganic polymeric coagulant (POFC)

#### The acute oral toxicity of POFC using healthy mice

The objective of the experimental study was to estimate the acute oral toxicity induced by the oral gavage administration of POFC in mice. All mice underwent a 1-week acclimatization phase following conventional husbandry procedures prior to the commencement of the research. The mice were housed in polypropylene cages measuring 45 cm × 24 cm × 15 cm. The animals were kept in a 12-h light and 12-h dark cycle, with temperature carefully maintained at 25 °C ± 2 °C. The mice were provided with unrestricted access to a standard pellet meal, water, and all essential resources throughout the duration of the trial. In order to adequately meet their nutritional requirements, the standard pellet meal consisted of 24% protein, 4% fat, 4.5% fiber, and 2% vitamins. A total of six healthy adult male albino mice weighing between 25 and 30 g were orally administered the test material via water at various dosage levels ranging from 100 to 5000 mg/kg body weight. The mice originated from the TodoreBilharz Research Institute (TBRI) located in Giza, Egypt, more specifically from the Schistosome Biological Supply Centre (SBSC).

The threshold hazardous dosage (LD_50_) of POFC on uninfected mice was calculated using WILBRANDT technique^34^. There are six mice each group, weighing between 20 and 25 g. Dimethylsulfoxide (DMSO) was administered orally to one group while the other was kept as the control. As of being delivered, various dosages are stated in mg/kg. Each group's toxic symptoms and fatality rate were noted. Each extract's LD_50_ was determined using the equation in Eq. (1):

1 $${{\text{LD}}}_{50}={{\text{D}}}_{{\text{m}}} - \frac{\mathrm{\Sigma Z x d}}{{\text{n}}}$$

where Dm: is the lowest dosage at which all animals in the group were killed; Z: is the mean of dead animals in two consecutive groups; d: is the constant factor between two consecutive groups; n: is the number of animals of each group; and Σ: is the sum of (Z × d).

#### Determination of cytotoxic effect on human normal fibroblast cell line (BJ1) for POFC

Mosmann method^35^ was used, where, 3-(4,5-dimethylthiazol-2-yl) 2,5-diphenyl tetrazolium bromide (MTT) was reduced to formazan, in order to assess cell viability. A biosafety class II level laminar flow cabinet (Baker, SG403INT, Sanford, USA) was used for all experimental procedures. These operations were completed under sterile conditions. The cells were suspended in DMEM-F12 medium with 1% L-glutamine, 10,000 U/ml potassium penicillin, 10,000 g/ml streptomycin sulphate, and 25 g/ml amphotericin B, and 1% antibiotic–antimycotic combination. The cell culture was maintained at 37 °C in an environment that contained 5% CO_2_. Cells were seeded on 96-well microtiter plates at a density of 10 × 10^3^ cells/well in fresh complete growth medium after a 10-day batch culture period. After that, the plates were placed in a water-jacketed carbon dioxide incubator (Sheldon, TC2323, Cornelius, OR, USA) that was heated to 37 °C and 5% CO_2_ for 24 h. The medium was withdrawn and new medium devoid of serum applied to the wells. The cells were incubated either alone (serving as the negative control) or in the presence of different POFC concentrations with range of 0.78–5000 ppm. After a second 48-h incubation period, 40 µl of MTT salt solution (2.5 g/ml) were applied to each well. After that, the cells were incubated for an additional 4 h at 37 °C with 5% CO_2_. Each well received 200 µL of 10% sodium dodecyl sulphate (SDS) in deionized water in order to halt the reaction and dissolve the formazan crystals. The plates were then held at 37 °C for the remainder of the day. A microplate multi-well reader (Bio-Rad Laboratories Inc., model 3350, Hercules, California, USA) was used to measure the samples' absorbance at 595 nm, with 620 nm serving as the reference wavelength. The SPSS 11 software's independent t-test feature was used to find statistically significant differences between the samples and the vehicle-containing negative controls (cells). The formula in Eq. 2 was used to calculate the percentage change in cell viability:

2 $$\mathrm{The\,\,percentage\,\,of\,\,viability}=\left(\frac{\mathrm{Reading\,\,of\,\,tested\,\,material }}{\mathrm{Reading\,\,of\,\,negative\,\,control}}-1\right)*100$$

A probit analysis was performed to determine the IC_50_ and IC_90_ using SPSS 11 program.

### Evaluation of antimicrobial properties of POFC

#### Antibacterial susceptibility testing (AST)

The antibacterial activity of the coagulant POFC was evaluated using the Kirby-Bauer agar diffusion test, employing both disc and well diffusion techniques. In this assay, six different types of pathogenic bacteria were tested; three Gram-negative strains (*E. coli* O157:H7 ATCC 35,150, *Pseudomonas aeruginosa* ATCC 10,145, and *Klebsiella pneumoniae* ATCC 13,883) and three Gram-positive strains (*Staphylococcus aureus* ATCC 43,300, *Bacillus subtilis* ATCC 33,234, and *Listeria monocytogenes* ATCC 35,152). To initiate the experiment, individual bacterial strains were inoculated into nutrient broth and incubated for 24 h at 37 °C. Subsequently, bacterial suspensions were prepared for each bacterial culture by combining the inoculum with sterilized test tubes containing 0.85% NaCl solution, resulting in suspensions with a density of 0.5 McFarland standard or 1.5 × 10^8^ CFU/mL^36^.

The bacterial strains under investigation were cultivated on Muller Hinton Agar (MHA) plates using a standardized inoculum in an aseptic manner. The disc-diffusion experiment was conducted under aseptic conditions. The sterile discs were subjected to air-drying using a laminar air flow cabinet subsequent to inoculate with 50 µL of the coagulant POFC at a concentration of 20 ppm. Negative controls were established by immersing discs in sterile, filtered water, while positive controls were represented by discs containing Vancomycin and Ciprofloxacin. Subsequently, the moistened discs were carefully placed onto the surface of the Mueller–Hinton agar (MHA) using aseptic forceps. Aseptic techniques were employed to create wells in the MHA agar medium, each measuring 6 mm in diameter. A sterile Cork borer was utilized to create these wells in a deep layer of the agar medium, for the purpose of conducting the well-diffusion test. POFC coagulant was introduced into each well using a 50 µL inoculation. Negative controls in this study consisted of wells containing sterile, filtered water, while positive controls were represented by Vancomycin and Ciprofloxacin. Subsequently, each plate was subjected to a 24-h incubation period at a temperature of 37 °C. The quantification of the zones of inhibition (ZOI) surrounding the discs and wells was performed using a Vernier calliper. The IC_50_ value was determined through the application of nonlinear dose–response modeling in the software Graph Pad Prism^37^. IC_50_ was calculated using nonlinear dose–response modeling in the Graph Pad Prism.

A macro-dilution test was used to evaluate the minimum inhibitory concentration (MIC) of the coagulant POFC. The tested coagulant had concentrations between 0 and 15 ppm. Antibiotic-containing media was used in a test tube as a positive control, whereas media containing bacterial inoculum were used as a negative control. To determine the MIC of POFC for each bacterial strain, a mixture of medium containing bacterial inoculum and different concentrations of POFC were used. After different retention times ranged from 5 to 20 min, 1 mL of the contents of each tube was transferred into sterile plates, and the proper amount of melted nutrient agar was added. Then, each plate was incubated for 24 h at 37 °C. By comparing the positive tubes with the negative (control) tubes, the MIC values were determined. The lowest concentration (MIC) at which no bacterial growth could be seen on the agar plates was determined. The findings were shown as the means derived from two independent replicates^38^.

#### Antifungal activity of POFC coagulant

Fungal strains *Aspergillus niger* and *Rhizopus oryzae* were sub-cultured on Potato dextrose agar (PDA) slants and incubated at 30 °C for 72 h, then these slants were maintained under sterilized paraffin oil as stock cultures. Antifungal activity of POFC was tested against *Aspergillus niger* and *Rhizopus oryzae* using Kirby-Bauer disc diffusion method^39^. One ml of stock fungal strains suspension was added to Petri dishes containing liquefied Potato dextrose agar (PDA) medium (45–50 °C). After solidification, filter paper disc (6 mm diameters) saturated with 50 μL of the tested POFC at concentration 20 ppm were put on the surface of media. Amphotericin B was used as positive control for the tested fungal pathogens. The plates containing PDA were incubated at 28 °C for 48 h. The diameters of inhibition zones (ZOI) around discs were recorded.

The minimum inhibitory concentration (MIC) was determined by testing the synthesized POFC at different concentrations 4–64 ppm which were added to sterilized media, then 1 ml of each fungal suspension were inoculated in each plate and incubated as mentioned previously. Amphotericin B (AMB), (Sigma, Chemical Co., USA) is tested as positive control was prepared in DMSO. The lowest concentration of POFC, which showed no fungal growth, defined as MIC. Every test was performed by duplicate^40^.

#### Antiviral activity of coagulant POFC

The antiviral activity of coagulant POFC was evaluated against highly pathogenic influenza viruses, namely SARS-CoV-2, MERS-CoV, and H5N1. Egypt/NRC-03/2020 SARS-CoV-2 (GSAID: EPI_ISL_430820)^41^, NRCE-HKU270 MERS-CoV (GenBank: KJ477103.2)^42^ and influenza H5N1 virus A/chicken/Egypt/B13825A/2017^43^, were isolated and propagated in The Center of Scientific Excellence For Influenza Viruses CSEIV at National Research Centre, Egypt. The beta-corona viruses MERS-CoV and SARS-CoV-2 were titrated using tissue culture infection dose 50 (TCID50) after being propagated in Vero E6 cells (ATCC No. CRL-1586), as previously reported in^43,44^. Serial dilutions of each virus were used to infect Vero-E6 monolayers in 96-well plates, which were then incubated for 72 h at 37 °C with 5% CO_2_. Infected cells were subjected to fixation with 3% paraformaldehyde and staining with crystal violet (0.1%). Reed and Munch equation was used to calculate the virus titer^45^.

Plaque titration assay was used to titrate influenza H5N1 as described by Kutkat et al*.*^43^. Vero E6 Cells were infected with different virus dilutions after being seeded as monolayers in six-well plates. At 1 h post infection, virus inoculum was removed and DMEM infection media supplemented with agarose were used as over-layer. Post incubation for 72 h post infection_,_ infected cell monolayers were then fixed and stained for visualization of plaques. Viral plaque forming unit (PFU/ml) was calculated as follows Eq. (3),

3 $$\frac{{\text{PFU}}}{{\text{ml}}}=\left({\text{N}}\right)\mathrm{number\,\,of\,\,plaques}*10*\mathrm{D }(\mathrm{the\,\,value\,\,from\,\,the\,\,dilution\,\,series})$$

##### CC_50_ cytotoxicity assay of coagulant POFC

VERO-E6 cells (cell isolated from the kidney of an African green monkey) were used to test the cytotoxic concentration^44,46^. The process of cell seeding was conducted using 96-well plates, where each well was allocated 100 µl of cell suspension at a concentration of 3 × 10^5^ cells/ml. The cells were subjected to treatment with varying concentrations of POFC in triplicate wells. Subsequently, the cells that had undergone treatment were subjected to an incubation period of 72 h following the infection. In order to prepare the dilutions of the coagulant, a series of serial dilutions were performed by creating twofold dilutions of the stock solution using cell culture growth media. The growth medium employed in this study consisted of Dulbecco's Modified Eagle's Medium (DMEM), which was supplemented with 10% fetal bovine serum (FBS) and 2% antibiotic–antimycotic solution. After fixing, cells were stained with crystal violet. At λ max 570 nm, absorbance was determined using Anthos Zenyth 200 rt plate reader. Using the following equation (Eq. 4), the cytotoxicity percentage of each dilution of POFC was tested in comparison to the untreated cells.

4 $$\mathrm{\% cytotoxicity}=\frac{\left(\mathrm{absorbance\,\,of\,\,cells\,\,without\,\,treatment}-\mathrm{absorbance\,\,of\,\,cells\,\,with\,\,treatment}\right)\mathrm{ \times }100}{\mathrm{absorbance\,\,of\,\,cells without\,\,treatment}}$$

concentration which shown 50% cytotoxicity (CC_50_) was calculated by plotting % cytotoxicity against sample concentration^35^.

##### Inhibitory concentration (IC_50_) of POFC coagulant

Serial dilutions of CC_50_ concentration for POFC were distributed in triplicates and treated with 100 TCID 50 concentrations of the viruses (SARS-CoV-2 and MERS-CoV)^44,46^. After 1 h of incubation, the virus/POFC mixtures were used to infect cell monolayers at room temperature (RT) for 1 h. After that, the infected cells were incubated for 72 h at 37 °C in an incubator with 5% CO_2_. Following the incubation period, the cells were fixed then stained using a 0.1% crystal violet solution. A multi-well plate reader was used to measure the color intensity at 570 nm after 100 μl of absolute methanol was added to dissolve the dye. The concentration needed to decrease the virus-induced cytopathic effect (CPE) by 50% in comparison to the virus control was calculated in order to determine the IC_50_ value for POFC. Nonlinear regression analysis of the normalized data was conducted using GraphPad Prism software.

##### Antiviral activities using Plaque reduction assay of coagulant POFC

Assay was conducted according to Hayden et al. and Kandeil et al.^47,48^. MDCK cells (10^5^ cells/ml) were cultured in a six well plate for 24 h at 37 °C. Low dose of POFC was prepared and incubated with 10^4^ PFU of the H5N1 virus for 1 h, and then the mix was used to infect monolayers of MDCK cells (ATCC) pre-plated in 6 well plates. At 72 h post infection, monolayers were then fixed and stained for visualization of plaques. MDCK cells that had not had any kind of viral treatment were infected using the control virus. Equation (5) explains how the number of plaques was counted and the percentage decrease in plaque production was calculated in relation to the control wells:

5 $$\mathrm{\%\,\,inhibition}=\frac{\mathrm{viral\,\,count }\left({\text{untreated}}\right)-\mathrm{viral\,\,count }({\text{treated}}}{\mathrm{viral\,\,count }({\text{untreated}})}*100$$

### Municipal wastewater treatment experiment using POFC as coagulant

The effect of POFC dosage on the coagulation performance was tested on municipal wastewater collected from wastewater treatment plant. Optimization of the chemical treatment is conducted using POFC for high strength municipal wastewater using Jar testing. The experimental parameters included dosages range of 10–60 ppm, rapid mixing at a speed of 300 rpm for a duration of 4 min, and subsequent sedimentation for 30 min. Next, the subsurface treated wastewater was collected in order to determine the efficiency of POFC as a coagulant/flocculant. The physicochemical and microbiological parameters were assessed using the American Standard Methods^49^. A control trial was carried out to evaluate the impact of the coagulation process without the inclusion of POFC. The analysis involved the determination of total coliform, fecal coliform, *E. coli, Salmonella*, and *Staphylococcus* spp. in the treated effluent of POFC-based coagulation. Detection and enumeration of total coliform, fecal coliform, and *E. coli* were done using the most probable number method (MPN/100 mL) according to standard methods 9221 B, E, F, respectively^49^. Salmonella was quantified on Bismuth sulfite agar according to standard methods 9260 B^49^. Also, *Staphylococcus* spp. was quantified according to standard methods 9213 B^49^. Moreover, the sludge after sedimentation was collected at the end of the mixing experiment and total coliform, fecal coliform, *E. coli,* fungi and yeasts were detected. Treatment efficiency was calculated according to the following Eqs. (6–7):

6 $$\mathrm{Log\,\,Reduction}={log C}_{i}- log {C}_{f}$$

7 $$Removal \left(\%\right)= \frac{{C}_{i}-{C}_{f}}{{C}_{i}} *100$$

where C_i_ and C_f_ are the microbial density in the municipal wastewater before and after the treatment (coagulation process).

### Ethical approval declarations

All experimental protocols were ethically approved by the International Animal Ethics Committee at the National Research Centre, and research involving animals was authorized under No. 20-049. The guidelines and regulations adhered to the ethical principles governing animal research. A statement to confirm that all methods are reported in accordance with ARRIVE guidelines (https://arriveguidelines.org).

## Results and discussion

### Preparation and characterization of inorganic polymeric coagulant POFC

**Figure 1:**
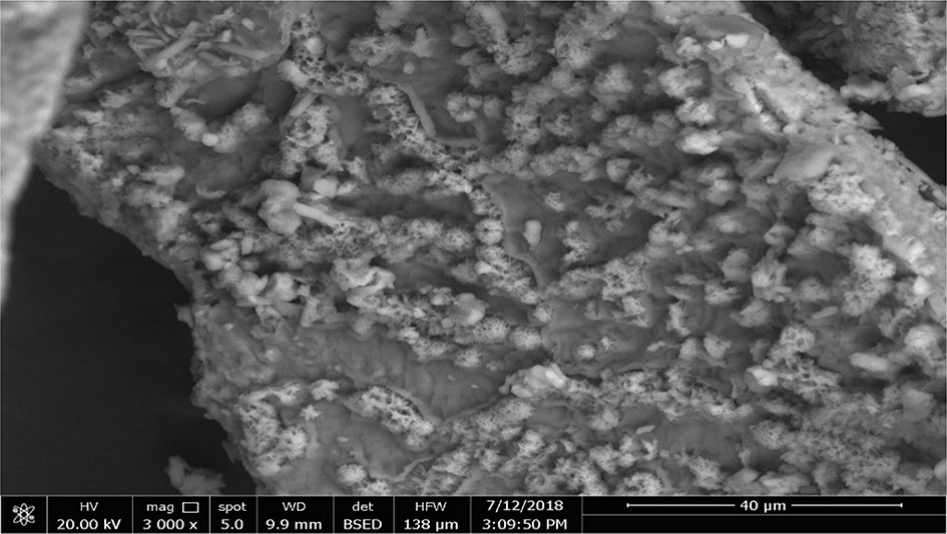
SEM of inorganic polymeric coagulant POFC-1-0.8

Preparation and structural analysis of POFC has been described in our previous publication^33^. The physical characteristics of POFC are used as coagulant/ flocculant aid which has a very high charge density (Zeta potential of 27.2 mV) to neutralize the negative charges present on the surface of the colloidal material, and its viscosity was accounted to be 10 centipoises. These properties allow good diffusion of the cationic charges around the particles that help to bridge, bind and strengthen the flocs and increase the settling rate. As well, its particle distribution has average of particle size 50 µm. The structural analysis of POFC by X-ray diffraction (XRD) and Fourier transform infrared (FTIR) spectroscopy were previously reported^33^. SEM images for POFC coagulant show the net-shaped structure and particle homogeneity as shown in Fig. 1. The net-structured coagulant is more favorable to coagulate colloidal particles and form bridge-aggregation among flocs^50^.

### Toxicity characteristic of POFC

#### The acute oral toxicity of coagulant using healthy mice

**Figure 2:**
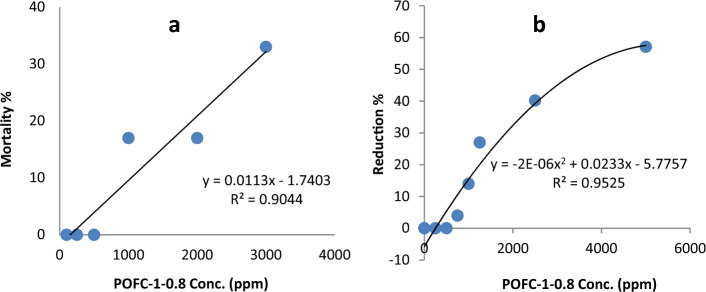
Toxic effect of different POFC-1-0.8 concentration on mice (**a**), human tumor cell lines (**b**).

The acute oral toxicity and mortality rate of POFC using healthy mice were recorded and shown in Fig. 2a and Table 1. Evidently, lethal rate of mice is neglected until administrated dose of 500 mg/kg. LD_50_ of POFC for mice was calculated according to WILBRANDT^34^ method and it was 3625 mg/kg.

**Table 1:** Lethal dose LD_50_ of POFC on healthy mice.

Dose (mg/kg b wt.)	Number of mice	Number of dead mice	Z	d	(Z) × (d)
100	6	0	0	100	0
250	6	0	0	150	0
500	6	0	0	250	0
1000	6	1	0.5	500	250
2000	6	1	1	1000	1000
3000	6	2	1.5	1000	1500
4000	6	3	2.5	1000	2500
5000	6	3	3	1000	3000

Σ (Z × d) = 8250 Σ (Z × d)/6 = 1375 mg/kg LD_50_ of POFC-1-0.8 = 3625 mg/kg.

#### Determination of cytotoxic effect on human normal fibroblast cell line (BJ1)

**Figure 3:**
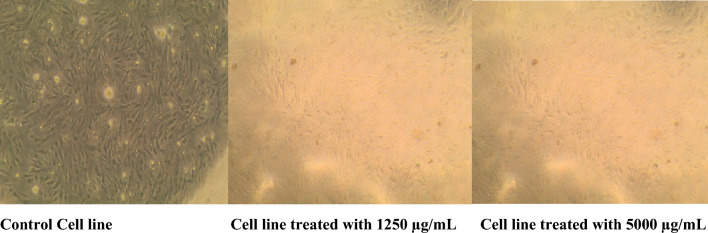
Cytotoxic effect of POFC-1-0.8 at different concentration on human normal fibroblast cell line (BJ1).

The sample was examined using the MTT assay at concentrations ranging from 50 to 5000 µg/mL (ppm) against the normal human epithelial cell line, BJ1 (normal Skin fibroblast). To assess the effect of POFC toxicity on epithelial cells, cell viability and cytotoxicity are investigated at different concentrations of POFC. Figure 2b demonstrated the influence of POFC on BJ11 cells viability. It was noted that human epithelial cell lines viability percentage decreased with increasing the concentration of POFC in comparing with control samples. The obtained results proved that POFC was non-toxic at 500 ppm. Meanwhile, it showed some weak toxicity at 1000 ppm, where 27% of cells death took place and the cytotoxicity increased to 70.1% at 5000 ppm. The calculated lethal concentration for 50% of the cells is 3266 (µg/ml) ppm. To confirm the cytotoxicity results of cell line after exposure to POFC, their microscopic examination was investigated. Figure 3 showed cell lines that treated with different concentrations. At POFC concentration of 1250 (µg/ml) ppm, epithelial cell lines count was partially influenced (See Figure 3). Meanwhile, the disappearance of epithelial cell lines was increased at higher POFC concentration of 5000 (µg/ml) ppm. Finally, the toxicological assay confirmed that POFC is eco-friendly and a green inorganic coagulant.

### Investigation of antimicrobial characteristics of inorganic polymeric coagulant

#### Antibacterial susceptibility testing

**Figure 4:**
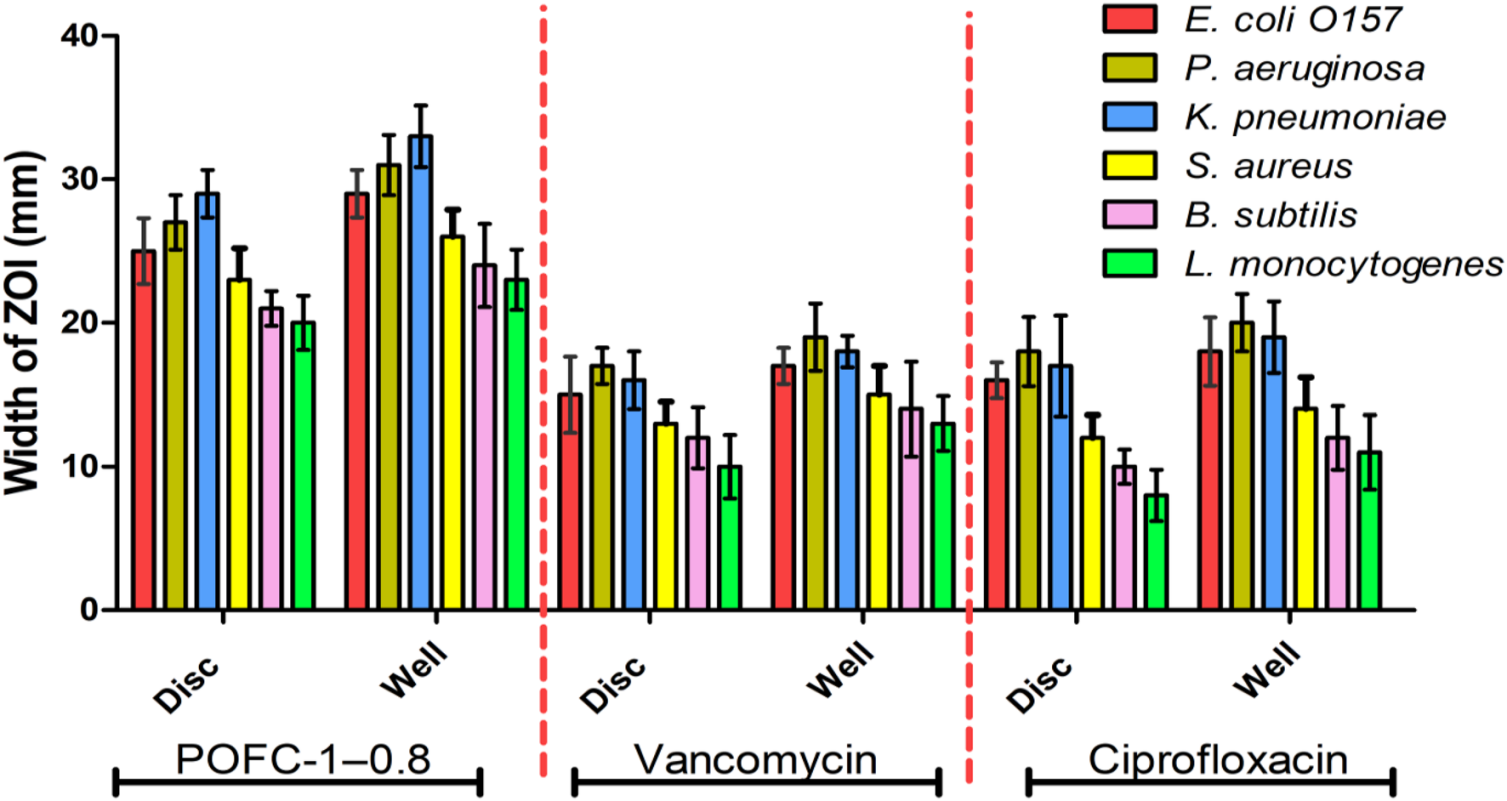
Inhibition zone diameters of POFC-1-0.8 against tested bacterial strains.

The antibacterial activity of POFC against *E. coli* O157, *P. aeruginosa, K. pneumoniae, S. aureus, B. subtilis,* and *L. monocytogenes* was tested using the disc and well diffusion assay. Results indicated that POFC at 20 ppm has a noticeably higher efficacy against Gram-negative bacteria than Gram-positive ones (Fig. 4). Gram-negative bacteria including *E. coli* O157, *P. aeruginosa*, and *K. pneumonia* showed inhibitory zones of 25, 27, and 29 mm using disc diffusion assay and 29, 31, and 33 mm using well diffusion assay, respectively. In parallel, the inhibitory zone values of POFC against the tested Gram-positive bacteria were 23, 21, and 20 mm using disc diffusion assay and 26, 24, and 23 mm using well diffusion assay for *S. aureus, B. subtilis,* and *L. monocytogenes,* respectively. Results indicated that the zone of inhibition (ZOI) diameters observed around the wells were greater than those around the discs, and this coincides with the findings of Abou Hammad et al.^51^. Moreover, it was found that the ZOI diameter produced by POFC was greater than that of the reference drugs vancomycin and ciprofloxacin. This suggests that POFC has a proficient inhibitory effect on bacteria.

**Figure 5:**
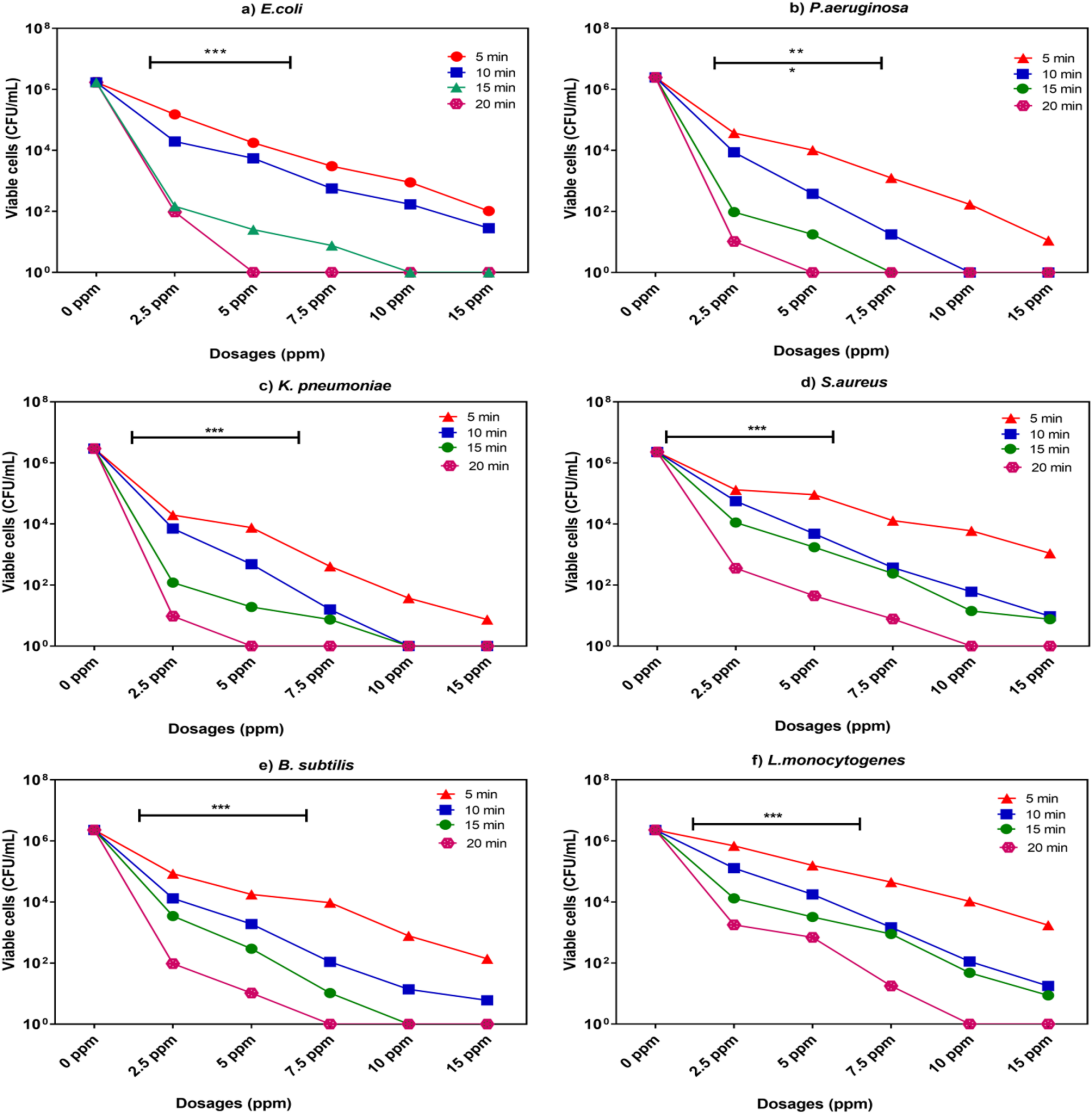
Minimum inhibitory concentration (MIC) values of POFC-1-0.8 against the examined bacterial species at different time intervals along with the results of the Two-way analysis of variance (*ANOVA*), where, **indicates a significant correlation (*p* ≤ 0.01), and ***indicates a high significant correlation (*p* ≤ 0.001).

Minimum Inhibitory Concentration (MIC) assay was used to verify the antibacterial potency of POFC using different concentrations (2.5–15 ppm) against the tested bacteria (Fig. 5). It was found that POFC inhibited the growth of all tested bacterial strains efficiently at varying MIC values. Within 20 min, the MIC values for *E. coli*, *P. aeruginosa*, and *K. pneumoniae* were 5 ppm and 7.5 ppm for *B. subtilis*. Additionally, the MIC for *S. aureus* and *L. monocytogenes* was 10 ppm. MIC values revealed that POFC exhibits greater potency against Gram-negative bacteria compared to Gram-positive bacteria. The observed variability in antibacterial efficacy may be attributed to variations in the composition of bacterial cell walls and the compound's capacity to permeate the cell membrane across different bacterial species^52^. Table 2 shows the IC_50_ values of POFC against the tested bacterial strains. The recorded values for *E. coli* O157, *P. aeruginosa, K. pneumoniae, S. aureus, B. subtilis*, and *L. monocytogenes* were 23.04, 10.89, 16.53, 24.31, 25.86, and 31.32 ppm, respectively, as presented in Table 2. The results of this study indicate that POFC exhibits a strong bactericidal effect against the microorganisms that were tested.

**Table 2:** The calculated IC_50_, Log IC_50,_ and R^2^ of POFC-1-0.8

	POFC-1-0.8		
	IC_50_ (ppm)	Log IC_50_ (ppm)	R^2^
*E. coli* O157	23.04	1.39	0.968
*P. aeruginosa*	10.89	1.05	0.947
*K. pneumoniae*	16.53	1.24	0.952
*S. aureus*	24.31	1.38	0.978
*B. subtilis*	25.86	1.53	0.983
*L. monocytogenes*	31.32	1.46	0.989

#### Antifungal activity of coagulant POFC

By measuring the zone of inhibition using the disc diffusion technique, the antifungal efficacy of POFC against *Aspergillus niger* and *Rhizopus oryzae* is shown in Table 3. The results showed that POFC had a noticeable inhibitory effectiveness at a concentration of 20 ppm against the tested black fungus strains. For *Aspergillus niger*, the zone of inhibition was 17 mm, while it was 24.6 mm for *Rhizopus oryzae* which is higher than that of reference standard antifungal (Amphotericin B). These results show that *Rhizopus oryzae* was more affected by POFC than *Aspergillus niger*. The antifungal potential of POFC at various doses was evaluated using MIC test. Table 3 shows that POFC has antifungal efficacy against *R. oryzae* with MIC value 8 ppm and has MIC value 32 ppm against *A. niger*. These results confirmed that POFC has a potential effect against black fungus especially against R. oryzae which show greater sensitivity to POFC than *A. niger*. The previous studies like those of Zahid indicated that the methanolic extract of *Euphorbia prostrata* has antifungal efficacy against *R. oryzae* and *A. niger*, with zone of inhibition measures of 18 mm and 9 mm, respectively^53^. Also, El-Newehy et al. studied the antimicrobial activity of the biocidal polymers against *Aspergillus niger*, *Cryptococcus neoforman,* *Candida albicans* and *Aspergillus flavus*^54^. They found that *A. niger* was the most affected fungus among the tested microorganisms with an inhibition zone of 19–21 mm for different biocides^54^. By comparing the obtained results of POFC against black fungus to the previous results stated in the literature; it is clear that POFC can be used as a powerful antifungal agent during wastewater treatment.

**Table 3:** ZOI diameters and Minimum inhibitory concentration of the synthesized POFC-1-0.8 against tested fungal pathogens.

Tested fungal pathogens	POFC-1-0.8	Standard ZOI using reference amphotericin B (mm)	POFC-1–0.8 MIC (ppm)
	ZOI (mm)		
*A. niger*	17	21	32
*R. oryzae*	24.6	21	8

#### Anti-coronaviruses activity of POFC

**Figure 6:**
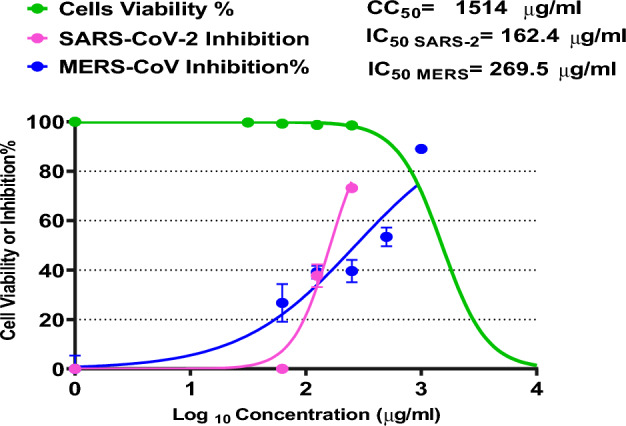
Anti-coronaviruses activity of POFC-1-0.8 against SARS-CoV-2 and MERS-CoV-2 using vero-E6 cells.

Antiviral activity of POFC against 2 diferent types of highly pathogenic beta-Coronaviruses (SARS-CoV-2 and MERS-CoV) was determined using inhibatory concentration IC_50_ assay. First, the in-vitro safety was detrmined on VeroE6 cells. The results reveal that POFC has high safety while the CC_50_ for kidney cells of an African green monkey was 757.2 µg/ml. Selectivety index (SI) is a ratio between cytotoxicity (CC_50_) and antiviral activity (IC_50_), which higher values of SI means higher effectivness. The IC_50_ of POFC against SARS-CoV-2 was 81.26 µg/ml, and the SI was 9.3, and The IC_50_ of POFC against MERS-CoV was 135 µg/ml (ppm), and the SI was 5.6, as demonstrated in Fig. 6. These results revealed the antiviral activity of POFC against MERS-CoV and SARS-CoV-2, with relatively high safety in vitro.

##### Anti-influenza virus activity of POFC

Plaque reduction assay was used to detemine the antiviral activity of POFC against influenza highly pathogenic H5N1 virus. POFC sucessfully inhibited the viral replication by 32.5% at low contentration of 12.5 µg/ml (Table 4).

**Table 4:** Antiviral activity of POFC-1–0.8 against high pathogenic avian influenza H5N1 activity using plaque reduction assay.

Sample	Contact time (min)	Dilution (µl/ml)	Virus control (PFU/ml)	Viral titer post-treatment (PFU/ml)	Viral inhibition (%)
POFC-1–0.8	60	12.5	4 × 10^5^	2.7 × 10^5^	32.5%

### Treatment of municipal wastewater and reduction of microbial loads in wastewater and the generated sludge using POFC

The abovementioned results prove that POFC has a great antibacterial, antifungal and antiviral effect at a concentration much lower than that its toxic level. POFC was applied in bench scale experiment on municipal wastewater samples to investigate its efficiency in reducing microbial loads in wastewater and in the generated sludge. Combined analysis of physico-chemical parameters is a useful procedure for monitoring the performance of POFC coagulant and the quality of wastewater and the generated sludge after treatment.

**Figure 7:**
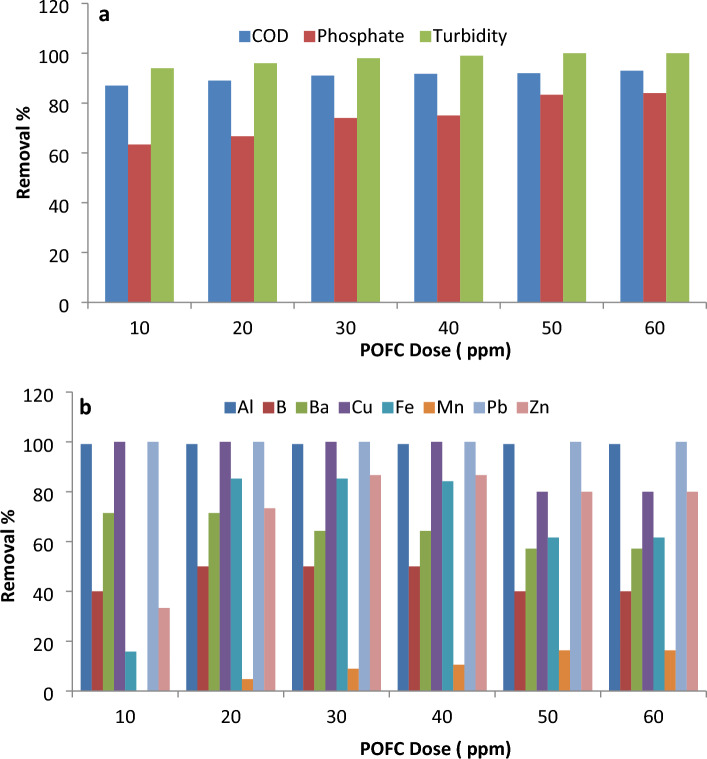
Treatment efficiency of municipal wastewater using POFC-based coagulation.

The coagulation-flocculation attempts for municipal wastewater were carried out using 10–60 ppm of POFC coagulant dose. The initial loads of chemical oxygen demand (COD) was 450 mg/L, total suspended solids (TSS) was 240 mg/L and turbidity was 160 NTU, phosphate was 6.0 ppm, and total phosphorus was 12 ppm. Figure 7 showed the results obtained for municipal wastewater treatment using different doses of coagulant-flocculant. The efficiency of removal for different pollutants from wastewater was shown in Fig. 7a. It was noticed that remarkable removals percentages for turbidity, phosphate, and COD from wastewater upon using POFC-based coagulation process. As shown, a slight reduction in COD, phosphate and turbidity achieved at doses higher than 30 ppm. Moreover, Fig. 7b showed interestingly removal for metals such as Al, Pb, Cu, Zn and Fe from real municipal wastewater after application of POFC-based coagulation process.

**Figure 8:**
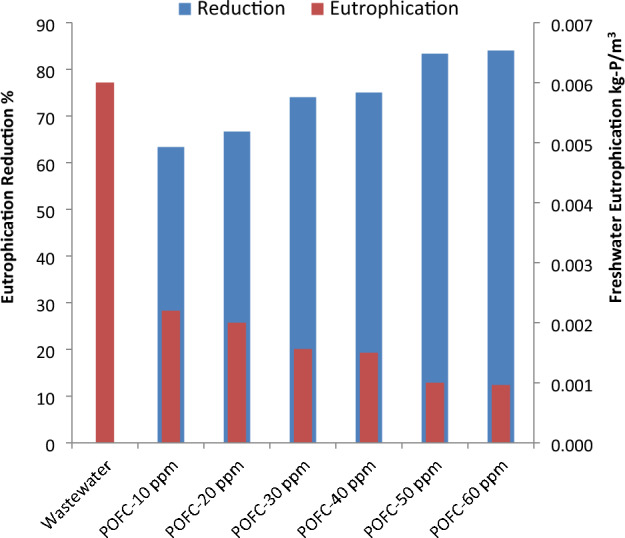
Freshwater eutrophication potential for wastewater after different POFC dose.

As controlling of phosphorous that discharged from treated municipal wastewater is a key factor in preventing eutrophication of surface water^55–57^. Therefore, the phosphorous removal rate is studied from treated municipal wastewater using POFC for decline eutrophication potential (Freshwater toxicity). Figure 8 showed the freshwater eutrophication (freshwater toxicity) potential for treated effluent with different POFC dose. It was remarkably noted that freshwater toxicity (kg-P/m^3^) was sharply decreased after application of POFC-based coagulation. The recorded eutrophication potentials are 0.006 and 0.001 kg P/m^3^ for municipal wastewater and treated effluents, respectively. The reduction rate of freshwater toxicity increased from 63 to 84% with increasing dose from 10 to 60 ppm. Conclusively, POFC-based coagulation is well-located option for declining freshwater eutrophication potential and freshwater toxicity.

**Figure 9:**
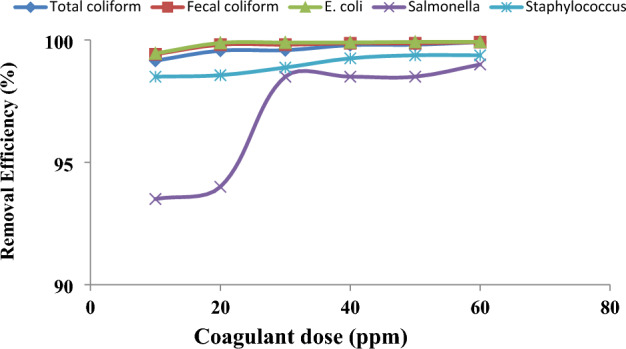
Removal efficiency of total coliform, fecal coliform, *E. coli*, *Salmonella* and *Staphylococcus* spp. from wastewater after additions of POFC-1-0.8 coagulant at different dosage rates (10–60 ppm).

The efficiency of the treatment in reducing the bacterial load from wastewater was assessed by estimating total coliform, fecal coliform, *E. coli*, *Salmonella* spp., and *Staphylococcus* spp. in municipal wastewater and in the treated effluent after POFC-based coagulation. The results revealed that the removal percentage of bacteria increased with increasing the POFC dosage from 10 to 60 ppm Fig. 9. The achieved removal rates range from 99.15 to 99.92% for total coliform, 99.42 to 99.92% for fecal coliform, 99.45 to 99.93% for *E. coli*, 93.50 to 99.00% for *Salmonella* spp., and 98.50 to 99.38% for *Staphylococcus* spp. (Table 5 and Fig. 9). By comparing the obtained results (removal efficiency of bacteria by POFC coagulant) to the literature values for removal of bacteria after the coagulation process, the obtained results were better than the results of the previous studies. Vunain et al.^58^ reported that removal efficiency of *E. coli* from the influent of Zomba wastewater treatment plant by conventional coagulants aluminum sulphate (alum) and ferric chloride at a dosage 15 g/L was 97% and 96%, respectively. Also, Akgul et al.^59^ reported that ferric chloride (PIX-311) reduced fecal coliform in digested sludge up to 82%.

**Table 5:** Bacterial cell populations of total coliform, fecal coliform, and *E. coli* in the initial wastewater, treated wastewater and produced sludge after treatment using different dosage of POFC-1-0.8

Bacterial count in initial sample	Total coliform (MPN-index/100 ml)		Fecal coliform (MPN-index/100 ml)		*E. coli* (MPN-index/100 ml)	
	1.1 × 10^7^		1.1 × 10^7^		2.0 × 10^5^	
Coagulant dose (ppm)	Treated wastewater	Slurry sludge	Treated wastewater	Slurry sludge	Treated wastewater	Slurry sludge
10	9.3 × 10^4^	7.5 × 10^5^	6.4 × 10^4^	4.8 × 10^5^	1.1 × 10^3^	1.5 × 10^3^
20	4.8 × 10^4^	4.8 × 10^5^	2.1 × 10^4^	2.1 × 10^5^	2.4 × 10^2^	1.5 × 10^3^
30	4.6 × 10^4^	4.8 × 10^5^	2.1 × 10^4^	2.0 × 10^5^	2.1 × 10^2^	7.0 × 10^2^
40	2.3 × 10^4^	2.8 × 10^5^	1.5 × 10^4^	2.0 × 10^5^	2.0 × 10^2^	3.0 × 10^2^
50	2.1 × 10^4^	2.4 × 10^5^	1.5 × 10^4^	1.5 × 10^5^	1.6 × 10^2^	3.0 × 10^2^
60	9.3 × 10^3^	2.1 × 10^5^	9.3 × 10^3^	7.5 × 10^4^	1.5 × 10^2^	3.0 × 10^2^

The efficiency of bacterial removal from municipal wastewater by POFC can be explained as a result of coagulation process which can remove microorganisms associated with suspended particles in wastewater through the processes of flocculation and sedimentation. In addition, POFC coagulant has a positive charge whereas the suspended particles in wastewater including bacteria are almost negatively charged and the mutual attraction are taken place due to the electrostatic interaction which established between bacterial cell wall and surface of the material as declared by Da Silva et al*.*^60^. Also, Gudz et al.^61^ stated that the thiol groups (–SH) of the proteins on the bacterial cell surface can react with iron ions leading to lysis of cells.

**Figure 10:**
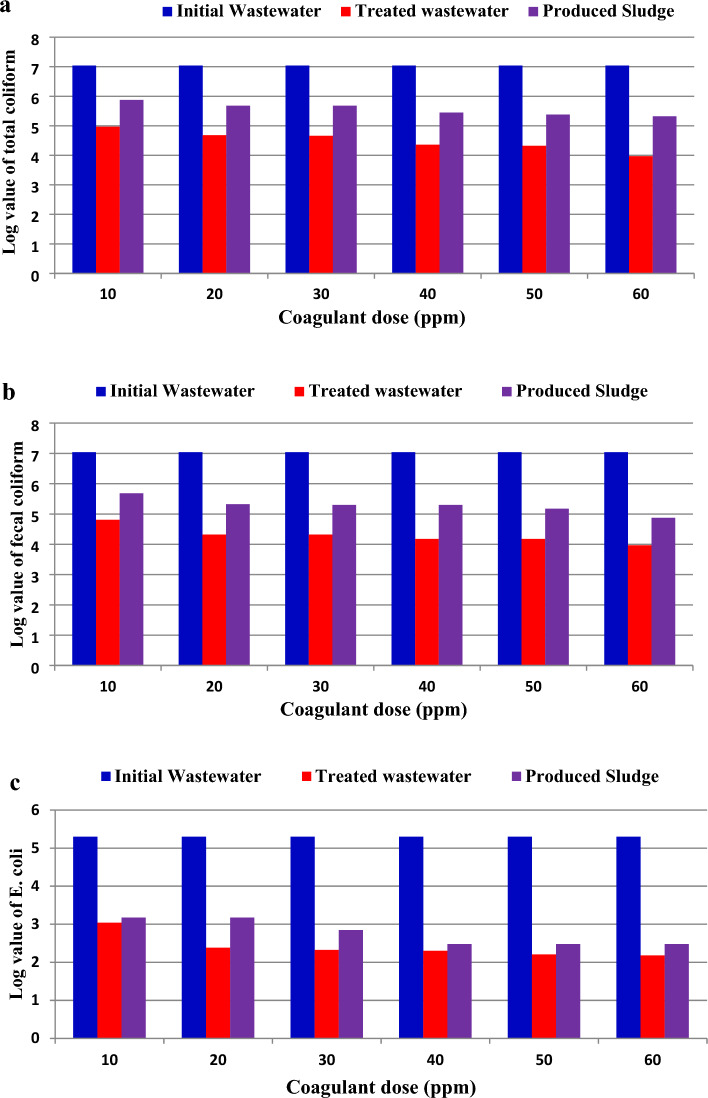
Log value of the density of (**a**) total coliform, (**b**) fecal coliform, and (**c**) *E. coli* (MPN/100ml) by multiple tube fermentation technique for municipal wastewater before and after treated and the generated sludge at different dosage of POFC-1-0.8 coagulant.

The sludge that produced after municipal wastewater treatment is recognized as a reservoir for pathogenic microorganisms because these microbes which include filamentous fungi and a high level of microbial clusters are transferred from wastewater to sludge during treatment processes^7,62^. In this study, the sludge which generated from the treatment of municipal wastewater with POFC was analyzed to identify chemical characteristics and detect the bacterial and the fungal load. The sludge that generated from chemical treatment is characterized and the data are shown in Table 6. The obtained data showed relatively high solid content, and sludge index. The sludge production to wastewater ratio is amounted to be 4.7%. Furthermore, total coliform, fecal coliform, and *E. coli* were also estimated in the sludge which generated from the wastewater after coagulation process by POFC (Fig. 10 and Table 5). The results indicated that the lowest bacterial load in the resulted sludge was achieved at a dosage 60 ppm of POFC. Also, it was observed that the bacterial load in the resulted sludge was higher than that in the treated wastewater at the same dose of POFC, but it was still less than the load found in the tested wastewater (the initial sample). This is due to the processes of flocculation and sedimentation/precipitation which happen during coagulation process. This is consistent with what Yang et al.^7^ stated that transferring pathogens from liquid to sludge during treatment is considered an important way to lessen pathogens in wastewater, so sludge is thought to be a reservoir of different pathogens. These results clearly indicated that the elimination of bacteria during treatment process is due to the dual effect of POFC, which acts as both a coagulant and an antibacterial agent. So, POFC can be used as a disinfectant against bacteria as well as it considered an effective coagulant during the pre-treatment of raw wastewater.

**Table 6:** Characteristics of generated sludge after chemical treatment for 1 L of municipal wastewater.

Parameter	Unit	
pH	–	8.2
Sludge volume	ml/L	47
Sludge weight	g/L	0.512
Sludge index	mL/L	92
Water Content	%	94.88
Solid content	%	5.12
Sludge production	%	4.7

To complete the analysis of the generated sludge after addition of different dosage of POFC, fungi and yeasts were detected in the generated sludge. Various types of filamentous fungi and yeasts were detected in the tested wastewater. Results recorded in Table 7 showed that there is a reduction in different fungi and yeasts after treatment by different dosage of POFC (10–60 ppm). At lower doses than 30 ppm of POFC, all types of fungi and yeasts existence were not affected. The removal for fungi and yeasts was observed at higher dose than 30 ppm of POFC (Table 7). The reduction efficiency of filamentous fungi and yeasts increased gradually from 5 to 84.9% with increasing the dosage of POFC from 30 to 60 ppm. The results indicated that POFC has a great effect on the existence of fungi and yeasts in the generated sludge after coagulation process for real municipal wastewater. This coincides with the antimicrobial activity results which proved that POFC has an effective antifungal effect. Therefore, POFC as a coagulant can be also used as an antifungal agent during the treatment of wastewater to overcome the fugal overgrowth which may cause fungal sludge bulking as stated by Feng et al.^62^. The antifungal effect of POFC may attributed to the effect of its cationic charge which enables it to bind strongly with the negatively charged microbial membrane surface^63,64^.

**Table 7:** Types of filamentous fungi and yeasts present in the initial municipal wastewater and the generated sludge after treatment by different dosage of POFC-1–0.8 coagulant (10–60 ppm).

Coagulant dose (ppm)	Types of fungi					
	*A.niger*	*A.flavus*	*A.fumigatus*	*Rhizopus* spp.	*Mucor* spp.	White yeast spp.
Initial	+	+	+	+	+	+
10	+	+	+	+	+	+
20	+	+	+	+	+	+
30	+	+	+	−	+	+
40	+	−	+	−	+	+
50	+	−	+	−	−	+
60	+	−	+	−	−	−

## Conclusion

The toxicity of POFC was assessed and employed as coagulant without additive for microbial deactivation of bacteria, virus and fungi. As well the efficiency for reducing the eutrophication impact of treated wastewater was investigated. The results confirm the low toxicity level of POFC on mice (LD_50_ of 3625 mg/kg) and human normal skin cells (IC_50_ of 3266 ppm). POFC shows a significantly high antibacterial activity against different bacteria with minimum inhibitory concentration (MIC) ranged from 5 to 7.5 ppm within 20 min. Moreover, POFC has antifungal efficacy against *Aspergillus niger* and *Rhizopus oryzae* with ZOI of 17 mm with MIC of 32 ppm with ZOI of 24.6 mm with MIC of 8 ppm, respectively. Furthermore, POFC exhibited a remarkable antiviral action against SARS-CoV-2 (IC_50_ = 81.26 µg/ml) and MERS-CoV (IC_50_ µg/ml), as well the highly pathogenic H5N1 influenza virus (with inhibition rate 32.5% at a concentration of 12.5 ppm). POFC demonstrated significant removal percentages of turbidity, phosphate, and COD at dosages ranging from 20 to 60 ppm. At a dose of 60 ppm, 84% reduction of eutrophication was achieved compared to municipal wastewater. Moreover, it exhibited a noteworthy removal percentage up to 99% or more for total coliform, fecal coliform, *E. coli*, *Salmonella* and *Staphylococcus* spp. at 60 ppm of POFC. Also, the bacterial density in the generated sludge after treatment was lower than that of the raw municipal wastewater due to antibacterial activity of POFC. Furthermore, the removal efficiency for filamentous fungi and yeasts in the resulted sludge steadily increased from 5 to 84.9% with POFC doses ranging from 30 to 60 ppm. Conclusively, POFC exhibits a high proficiency for eutrophication reduction and microbial deactivation for wastewater and sludge. POFC could be a promising coagulant-flocculant for decontamination of municipal wastewater treatment processes with high potency of eutrophication reduction. The future outlook is to investigate the performance of POFC for sludge treatment. The cost analysis for POFC-based treatment will be further carried out.

## Data Availability

The corresponding author can provide the datasets used and/or analyzed for this study upon reasonable request.
